# The association between cardiorespiratory fitness and resting‐state functional connectivity in adults with Down syndrome

**DOI:** 10.1002/alz.70297

**Published:** 2025-05-20

**Authors:** Julianne G. Clina, Jessica C. Danon, Brian C. Helsel, Rebecca J. Lepping, Laura E. Martin, Joseph R. Sherman, Morgan G. Brucks, Joseph E. Donnelly, Lauren T. Ptomey

**Affiliations:** ^1^ Department of Internal Medicine University of Kansas Medical Center Kansas City Kansas USA; ^2^ Department of Neurology University of Kansas Medical Center Kansas City Kansas USA; ^3^ Department of Population Health University of Kansas Medical Center Kansas City Kansas USA

**Keywords:** Alzheimer's disease, Down syndrome, fitness, functional connectivity, intellectual disabilities, physical activity

## Abstract

**INTRODUCTION:**

Resting‐state functional connectivity (FC) of the default mode network (DMN) is linked to Alzheimer's disease in people with Down syndrome (DS). In adults without DS, cardiorespiratory fitness is associated with DMN FC; however, this has not been unexplored in DS.

**METHODS:**

This analysis used baseline data from an intervention in adults with DS. Resting‐state functional magnetic resonance imaging measured connectivity from the posterior cingulate cortex seed to DMN nodes. Fitness was measured by the maximal treadmill test. Pearson correlations and linear regressions were used to examine the associations between fitness and FC.

**RESULTS:**

Data from 40 adults with DS (26.0 years, 58% female) showed fitness was associated with overall DMN connectivity (*r* = 0.472, *p* = 0.004) and medial prefrontal cortex connectivity (*r* = 0.431, *p* = 0.010). The association between fitness and DMN FC remained significant after adjustment for age and sex (β = 0.0072, *p* = 0.04).

**DISCUSSION:**

Fitness may be associated with DMN FC in DS.

**Highlights:**

- In adults with DS, cardiorespiratory fitness was associated with overall DMN connectivity, which remained significant after adjusting for age and sex.
- No associations were found between moderate to vigorous physical activity and DMN connectivity.
- Increasing fitness may be a therapeutic strategy for AD prevention or delay in DS.

## 1 BACKGROUND

Down syndrome (DS), or trisomy 21, is the most common genetic cause of intellectual disability.^1^ Over the last several decades, the median life expectancy for persons with DS has increased from 4 years in the 1950s to approximately 60 years in the 2020s^2^, ^3^, ^4^ (20th percentile: ∼35 years; 80th percentile: ∼62 years),^4^ largely due to improvements in medical care for persons with DS. This increase in life expectancy has also illuminated several age‐related challenges for persons with DS. For example, virtually all persons with DS will develop Alzheimer's disease (AD)‐associated pathology.^5^, ^6^, ^7^ The high incidence of AD in DS is a result of the overproduction of amyloid beta (Aβ) from the additional copy of the amyloid precursor protein found on chromosome 21, which accelerates neurodegeneration, oxidative stress, plaque deposition, and early development of AD‐like pathology.^6^, ^8^, ^9^ Research suggests that deposition of Aβ occurs decades earlier in adults with DS compared with typically developed adults^10^, ^11^, ^12^ such that the lifetime incidence of dementia is 90%,^8^, ^10^ with a median age at onset of 53.8 years. A recent review found significant individual variability in the onset of AD in persons with DS (25 years),^13^ suggesting that even in the context of amyloid positivity, the risk of dementia is modifiable.

The default mode network (DMN) is a group of interconnected brain regions that show increased activity in the absence of external stimuli or rest, such as daydreaming, autobiographical memory, or introspection,^14^ and has been observed to be disrupted during neurodegenerative disorders such as AD.^15^, ^16^ Regions that make up the DMN include the prefrontal cortex, angular gyrus, precuneus/posterior cingulate cortex (PCC), and temporal areas.^14^, ^17^ In a longitudinal study using data from individuals without DS in the UK Biobank (*n* = 81 individuals that developed dementia, *n* = 1030 matched controls), reduced connectivity of the DMN predicted future incidence of dementia.^17^ Recent research in persons with DS suggests that lower resting‐state functional connectivity (FC) in the DMN is an early AD biomarker and may be associated with greater Aβ accumulation in the inferior parietal cortex.^18^ Given the association between DMN connectivity and AD and its potential as a preclinical biomarker of AD in persons without DS, it is important to investigate this association in persons with DS and identify factors associated with maintaining connectivity.

**RESEARCH IN CONTEXT**

1. **Systematic review**: The authors reviewed the literature using traditional databases (e.g., PubMed). In adults with DS, resting‐state FC of the DMN is a correlate for age‐related decline in brain function and AD in DS. In adults without DS, cardiorespiratory fitness has been associated with better DMN FC, but this has not been investigated in DS populations. These relevant sources are appropriately cited.
2. **Interpretation**: In adults with DS, cardiorespiratory fitness, but not moderate to vigorous physical activity engagement, was associated with overall DMN connectivity even after adjusting for age and sex. These results align with non‐DS populations and reinforce the importance of fitness for AD‐related outcomes in persons with DS.
3. **Future directions**: The manuscript proposes fitness as a potential therapeutic target for AD prevention or delay in populations with DS. Future work should investigate whether a change in fitness yields improvements in DMN FC.

In typically developed adults, modifiable lifestyle factors, such as moderate to vigorous physical activity (MVPA) and cardiorespiratory fitness, are neuroprotective and may reduce the risk for or delay the onset of AD.^19^, ^20^, ^21^ MVPA is a measure of physical activity participation and has been associated with AD outcomes. Leisure‐based physical activity in midlife assessed via self‐report questionnaire was associated with lower odds of developing AD (*n* = 1449, population‐based survey with a mean 21‐year follow‐up).^22^ Further, in a sample of 308 adults without DS, higher physical activity participation across 10 years was associated with posterior DMN connectivity and perfusion.^23^

Measures of cardiorespiratory fitness, including peak oxygen intake during exercise (VO_2peak_), have also demonstrated associations with AD outcomes in the general population. Cardiorespiratory fitness has been associated with improved cerebral artery compliance and greater brain volume, suggesting that those with higher fitness may have more efficient brain metabolism and cerebral oxygen utilization.^24^ Studies implementing functional magnetic resonance imaging (fMRI) have demonstrated that higher cardiorespiratory fitness is associated with superior brain activation of the anterior cingulate, lateral prefrontal, and lateral parietal cortex across a variety of cognitive tasks.^21^ Cardiorespiratory fitness has been associated with increased FC of the DMN, specifically in older adults.^25^ However, we are unaware of previous research examining the association between these lifestyle factors and FC in adults with DS. Additionally, since MVPA and cardiorespiratory fitness capture distinct aspects of physical activity and fitness and do not always correlate, it is important to investigate these separately as predictors of DMN connectivity in this population.

The purpose of this study was to measure the association of MVPA and cardiorespiratory fitness with the FC of the DMN, measured using fMRI, in adults with DS. It was hypothesized that higher cardiorespiratory fitness and higher MVPA would be associated with higher levels of FC.

## 2 METHODS

This is a cross‐sectional analysis using baseline data from a physical activity trial in adults with DS without dementia.^26^ Participants attended a laboratory visit for assessments of cardiorespiratory fitness and then completed a MRI scan within 30 days of the fitness assessment. All data were collected between January 2020 and November 2022. This study was approved by the University's Institutional Review Board. Informed consent and assent were obtained from participants and their parents/legal guardians prior to data collection.

### 2.1 Participants

Participants in the original clinical trial (parent study; *n* = 81) were adults (≥18 years of age) with DS who were living at home with a parent or guardian or in a supported living environment with a caregiver who agreed to serve as a study partner. Additional inclusion criteria were mild to moderate intellectual disabilities as determined by the caregiver, sufficient functional ability to understand directions, ability to communicate through spoken language, ability to participate in physical activity and walk 10 feet unassisted, and having internet access in the home. Participants were excluded if they had dementia as determined by the Dementia Screening Questionnaire for Individuals with Intellectual Disabilities (DSQIID),^27^ participated in a regular exercise program (i.e., ≥20 min per day, ≥3 days per week.), had a serious medical risk, such as cancer or a recent cardiac event as assessed by the primary healthcare provider, were unable to participate in MVPA, or had any contraindications for MRIs. Participants were recruited using flyers, listservs, and social media posts by local organizations that provide services to adults with DS (e.g., DS societies, day service programs, clinics, and community developmental disability organizations funded by Medicare). Participants could be living with a family member, alone or with roommates, or in a supported group living environment. Caregivers of prospective participants were asked to contact the study coordinator, who answered questions about the study and administered the initial participant eligibility screener, which included questions about the participant's functional ability, communication skills, and the DSQIID. A home visit or video conference meeting was scheduled with those remaining interested and potentially eligible to determine final eligibility and to obtain consent.

### 2.2 Physical activity assessment

MVPA was assessed using an ActiGraph wGT3XBT tri‐axial accelerometer (ActiGraph LLC, Pensacola, FL, USA) worn on the non‐dominant hip at the anterior axillary line during waking hours for 7 consecutive days. Vertical axis data aggregated over 60‐s epochs from the ActiGraph devices were initialized and downloaded using Actilife software version 6.13.3 (ActiGraph LLC, Pensacola, FL, USA). Non‐wear time was defined as at least 90 consecutive minutes of zero counts, with an allowance for 1 to 2 min of movement between zero and 100 counts/min.^28^ Counts ≥20,000·/min were considered spurious.^29^ A wear time ≥8 h on at least 3 days including 1 weekend day was required for inclusion in the analysis. Vertical axis ActiGraph cut points used for adults in the 2003 to 2004 and 2005 to 2006 cycles of National Health and Nutrition Examination Survey (NHANES) were used to classify minutes of MVPA (≥3 METs; ≥ 2020 counts/min).^30^, ^31^

### 2.3 Cardiorespiratory fitness assessment

Cardiorespiratory fitness (VO_2 Peak_) measured in mL/kg/min was assessed using a maximal treadmill test, following the protocol described by Fernhall et al. developed for adults with DS.^32^ Expired O_2_ and CO_2_ were measured using indirect calorimetry (ParvoMedics TrueOne 2300, Salt Lake City, Utah, USA), which was calibrated with known volume and gas concentrations prior to each test according to manufacturer recommendations. Values were averaged over 15‐s intervals across the treadmill protocol. The exercise test was terminated if participants were unable to maintain the treadmill speed or requested that the test be stopped at any time during the treadmill protocol. Otherwise, tests were terminated when participants achieved two or more of the following criteria: (1) volitional exhaustion, (2) a plateau in VO_2_, that is, < 150 mL/min or heart rate (HR) < 2 beats/min with increased work rate, (3) a HR within five beats/min of HR_Peak_ predicted using the formula of Fernhall et al.,^32^ and (4) a respiratory exchange ratio ≥ 1.0. Only participants who achieved a respiratory exchange ratio ≥ 1.0 or had a HR within 10 beats/min of their HR_Peak_ were included in the analysis.

### 2.4 Neuroimaging

Scanning was performed on a 3T Siemens Skyra scanner (Siemens, Erlangen, Germany) fitted with a 20‐channel head coil. Following automated scout image acquisition and shimming procedures to optimize field homogeneity, participants completed a 40‐min scan, which included structural (MPRAGE), resting‐state functional MRI (rsfMRI),^33^ diffusion tensor imaging, and arterial spin labeling (ASL) sequences.^34^ Participants were able to terminate the scan at any time during the procedure. rsfMRI was performed using a gradient echo multiband EPI sequence (repetition time/echo time  =  1000/40 ms, multiband factor = 8, slice thickness  =  2.5 mm, in‐plane resolution  =  2.5 mm, 64 slices, 480 measurements), with scans lasting 8 min.^35^, ^36^, ^37^ Data preprocessing and statistical analyses were performed using analysis of functional neuroimages (AFNI)^38^ following their standard recommendations. Preprocessing scripts were generated using the command afni_proc.py and included slice time correction, motion correction, alignment, spatial smoothing, and normalization. For each participant, MPRAGE data were skull stripped and normalized to standard Montreal Neurological Institute (MNI) space by non‐linear warping (AFNI command @SSwarper). rsfMRI images were realigned to the minimum outlier to correct for motion. Data with motion >0.2 mm within a volume were censored. Outlier and motion volumes were censored immediately before the data with motion, as recommended by AFNI. After censoring, the duration of rsfMRI was a mean of 245 s (range: 26 to 478 s). All available data were included, as previous work demonstrated that censoring did not bias group analyses.^39^ Predicted time course was constructed from nuisance variables, including six motion parameters (translation and rotation around *x*, *y*, *z* axes), average ventricle signal and white matter signal from FS masks, and physiological regressors of HR and respiration. HR and respiration were measured in the scanner with a Biopac MR comparable respiration belt and pulse plethysmograph (Biopac MP150 and AcqKnolwedge 5 Software, BIOPAC Systems, Inc., Goleta CA, USA). Nine percent of participants did not have usable physiological regressors, and their data were processed without that correction. This nuisance time course was subtracted from each voxel, resulting in a residual time course for each voxel. The residual time course was then smoothed with a 4‐mm full width half maximum Gaussian kernel, resampled to a 2.5 × 2.5 × 2.5 mm grid, and transformed to MNI space.

The PCC seed^40^ was used to identify the DMN regions of interest (ROIs) within the current sample. A spherical PCC seed with a 5‐mm radius was created with the central point at (*x*, *y*, *z* = 0, −56, 28). The average time course was extracted from the PCC seed region, and a regression was run to calculate the Pearson correlation with the seed at each voxel across the brain. This correlation coefficient was then converted to Fisher *z‐transformed* values (*z*‐scores) for each participant for group analyses.

**FIGURE 1:**
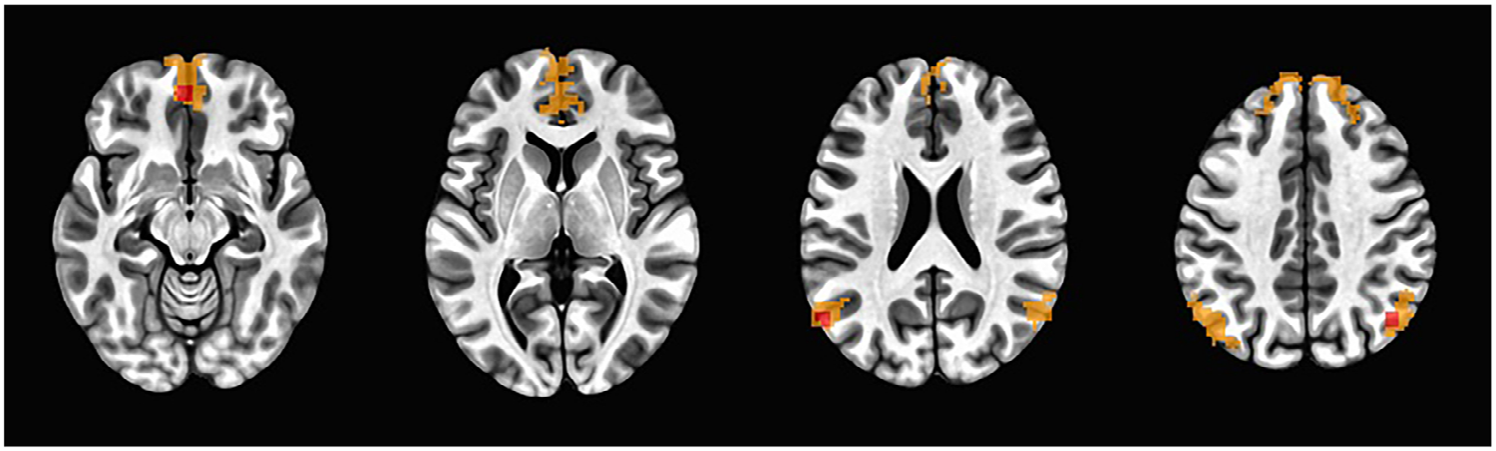
Axial view of DMN map on ROI seeds included in analyses. DMN, default mode network; ROI, region of interest.

A whole‐brain 3dttest++^38^ was run using z‐scores to determine ROIs if significant connectivity to the PCC seed to index DMN connectivity within the sample. The resulting DMN connectivity map at *p *< 0.01 false discovery rate (FDR) corrected (Figure 1) was used to identify DMN specific ROIs. The analysis identified four ROIs showing significant connectivity to the PCC seed. The ROIs included the full DMN map, medial prefrontal cortex (MPFC *x*, *y*, *z* = −3, 51, −12), left parietal cortex (LPAR *x*, *y*, *z* = −58, −67, 25), and right parietal cortex (RPAR *x*, *y*, *z* = 45, −67, 38). Next, the peak voxel within each ROI was identified, and spherical seeds with a 5‐mm radius were created around the peak voxel. The average *z*‐score was extracted from the ROI seeds as well as the full DMN map using 3dROIstats and exported for group analysis to examine the relationship between FC within the DMN (i.e., *z*‐score for each ROI) and cardiorespiratory fitness.

### 2.5 Statistical analysis

Sample characteristics were described as mean ± standard deviation or frequency (percentage) for demographic (i.e., age, race, ethnicity, level of support), anthropometric (i.e., height, weight, body mass index), accelerometry (MVPA), and cardiorespiratory fitness (VO_2Peak_) measures. Main predictors (i.e., VO_2peak_ and MVPA) and outcomes (i.e., FC measures) were evaluated for normality using visual inspections (i.e., histograms and Q‐Q plots) and Shapiro–Wilk tests. VO_2peak_ overall DMN connectivity and PCC to MPFC were normally distributed based on the visual inspection and Shapiro–Wilk tests. All other measures (MVPA and PCC to the left and right parietal cortex) were slightly right‐skewed but approached a normal distribution, so parametric tests were used in this analysis. Pearson correlations were used to describe the association between MVPA or cardiorespiratory fitness and FC from the PCC seed to each DMN region indexed by the *z*‐score and the overall DMN map. Linear regressions were used to further explore the impact of MVPA or cardiorespiratory fitness on DMN connectivity in adults with DS while including age and sex in the model. Pearson correlation was also used to evaluate the association between MVPA and VO_2peak_ in this sample. R version 4.2.2 was used for this analysis.^41^

## 3 RESULTS

### 3.1 Participant characteristics

A total of 40 adults with DS from the parent study opted in and completed the fMRI used in this analysis. Of these 40 participants, 35 successfully completed the cardiorespiratory fitness assessment, and 31 returned valid accelerometer data defined by the criteria outlined previously. Characteristics of the sample are presented in Table 1. The average age of the participants was 26.0 ± 7.8, ranging from 18 to 45 years. The majority of participants were non‐Hispanic White (83%), with an average body mass index of 33.2 ± 6.6 kg/m^2^, average MVPA of 13.4 ± 14.8 min per day, and baseline VO_2peak_ of 20.2 ± 4.2 mL/kg/min. There was no association between MVPA and VO_2peak_ in this sample (*r* = 0.02, *p *= 0.89).

**TABLE 1:** Sample characteristics of adults with Down syndrome.

	Overall *n* = 40
Age (years)	26.0 ± 7.8
*Gender*	
Male	17 (43%)
Female	23 (58%)
*Race*	
American Indian	0 (0%)
Asian	0 (0%)
Pacific Islander	0 (0%)
African American	5 (13%)
Caucasian	33 (83%)
Mixed race	2 (5.0%)
*Ethnicity*	
Not Hispanic or Latino	38 (95%)
Hispanic or Latino	2 (5.0%)
*Level of support*	
Mild	30 (75%)
Moderate	10 (25%)
Heart condition present	25 (63%)
Weight (kg)	75.1 ± 15.7
Body mass index	33.2 ± 6.6
MVPA	13.4 ± 14.8
VO2 (mL/kg/min)	20.2 ± 4.2

Abbreviations: MVPA, moderate to vigorous physical activity; VO2, volume of oxygen.

^1^Mean ± SD; n (%).

### 3.2 Associations between cardiorespiratory fitness and MVPA and FC

**FIGURE 2:**
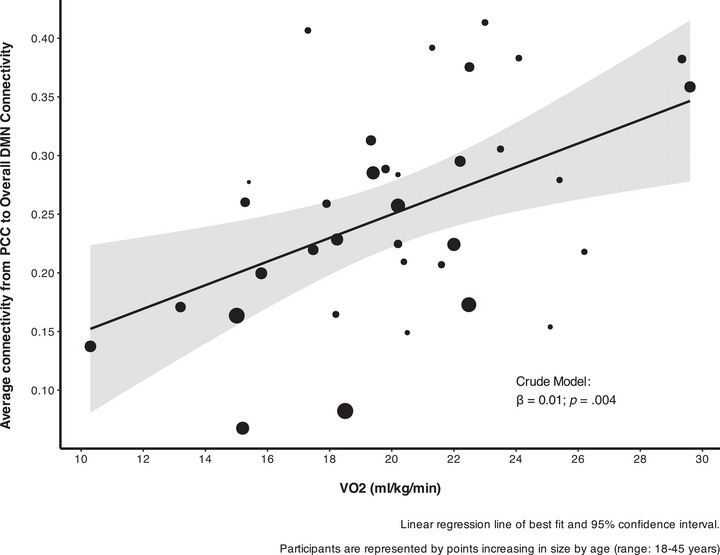
Association between average PCC to overall DMN mask connectivity and VO2 peak using linear regression adjusted for age and sex. Depicts the line of best fit from linear regression and 95% confidence interval. Participants are represented by points increasing in size by age (range: 18 to 45 years). β, beta estimate of linear regression; DMN, default mode network; PCC, posterior cingulate cortex; VO2, volume of oxygen.

**FIGURE 3:**
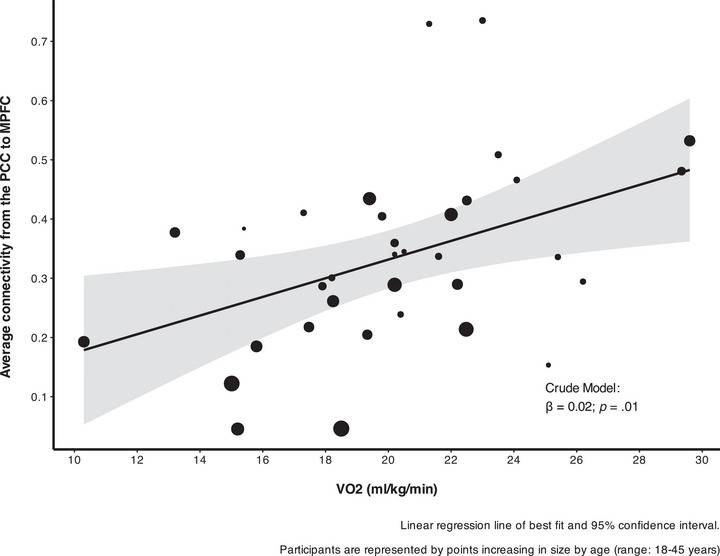
Association between average PCC to MPFC connectivity and VO2 peak using linear regression adjusted for age and sex. Depicts line of best fit from linear regression and 95% confidence interval. Participants are represented by points increasing in size by age (range: 18 to 45 years). β, beta estimate of linear regression; DMN, default mode network; MPFC, medial prefrontal cortex; PCC, posterior cingulate cortex; VO2, volume of oxygen.

Pearson correlations were used to evaluate the correlation between VO_2peak_ and MVPA on overall DMN connectivity and connectivity between the PCC seed and each ROI seed (Table 2). In this unadjusted model, VO_2peak_ was associated with increased overall DMN connectivity (*r* = 0.472, *p* = 0.004) and connectivity between the PCC seed and MPFC (*r* = 0.431, *p* = 0.010) but not the left or right parietal cortices. To determine whether this association was isolated to the specific MPFC ROI, we also conducted an exploratory whole‐brain analysis of connectivity of the PCC seed and VO_2peak._, which did not yield statistically significant results. There were no significant associations between MVPA and overall DMN connectivity or connectivity of any individual ROI seed. When adjusting for age and sex using linear regression (Table 3), VO_2peak_ remained a significant predictor of overall DMN connectivity (Figure 2) (β = 0.0072, *p* = 0.04). However, the association between VO_2peak_ MPFC connectivity (Figure 3), was just above the threshold for significance (β = 0.011, *p* = 0.07). There were no significant associations between VO_2peak_ and connectivity with the left and right parietal cortices or MVPA with the FC of any region in the adjusted models.

**TABLE 2:** Resting‐state functional connectivity Pearson correlations with cardiorespiratory fitness and MVPA.

	VO2 peak			MVPA		
	*N*	*r*	*P* value	*N*	*r*	*P* value
Overall DMN connectivity	35	0.472	0.004	31	−0.077	0.682
PCC to MPFC	35	0.431	0.010	31	−0.023	0.901
PCC to LPAR	35	0.230	0.185	31	−0.069	0.713
PCC to RPAR	35	0.188	0.280	31	−0.023	0.903

*Note*: Pearson correlation coefficients (r) and *p* values presented for associations between VO2 peak and MVPA with resting‐state FC across various brain regions. Significant correlations are indicated by *p* < 0.05.

Abbreviations: DMN, default mode network; LPAR, left parietal cortex; MPFC, medial prefrontal cortex; MVPA, moderate to vigorous physical activity; PCC, posterior cingulate cortex; RPAR, right parietal cortex; SE, standard error.

**TABLE 3:** Impact of cardiorespiratory fitness and MVPA on resting‐state FC using linear regression.

Region	β	SE	*p* value	F‐statistic	Adjusted *R* ^2^
**Predictor: VO2 (ml/kg/min)**					
Overall DMN connectivity	7.2e‐03	3.4e‐03	0.04	F(4, 31) = 5.46	0.28
PCC to MPFC	1.1e‐02	5.8e‐03	0.07	F(4, 31) = 5.48	0.28
PCC to LPAR	3.1e‐03	5.8e‐03	0.59	F(4, 31) = 1.92	0.08
PCC to RPAR	5.2e‐03	7.4e‐03	0.49	F(4, 31) = 0.70	−0.03
**Predictor: MVPA (min/day)**					
Overall DMN connectivity	−1.1e‐03	1.0e‐03	0.30	F(4, 27) = 3.03	0.17
PCC to MPFC	−1.6e‐03	1.9e‐03	0.40	F(4, 27) = 3.46	0.2
PCC to LPAR	−1.5e‐03	1.6e‐03	0.35	F(4, 27) = 2.14	0.1
PCC to RPAR	−9.1e‐04	2.1e‐03	0.67	F(4, 27) = 0.50	−0.05

*Note*: Linear regressions were adjusted for age and sex. The table presents regression coefficients (β) and SE of the regression coefficients for each predictor (VO2 and MVPA) across different brain regions. Significance indicated by *p* < 0.05.

Abbreviations: DMN, default mode network; FC, functional connectivity; LPAR, left parietal cortex; MPFC, medial prefrontal cortex; MVPA, moderate to vigorous physical activity; PCC, posterior cingulate cortex; RPAR, right parietal cortex; SE, standard Error; VO2, volume of oxygen.

## 4 DISCUSSION

The purpose of this analysis was to evaluate the association between MVPA and cardiorespiratory fitness on DMN connectivity in adults with DS. Results from this study indicate that increased cardiorespiratory fitness is associated with higher overall DMN connectivity and connectivity of the MPFC and PCC. The association between overall DMN connectivity and cardiorespiratory fitness remained significant even after adjusting for age and sex; however, this association was just above the significant threshold after adjustment for covariates with the MPFC connectivity (*p* = 0.07). MVPA was not associated with connectivity of any DMN node, and there was no association between MVPA and cardiorespiratory fitness in this sample.

Findings related to cardiorespiratory fitness align with current literature in populations without DS, which has identified that cardiorespiratory fitness is a predictor of DMN connectivity.^25^, ^42^ One such study included young adults (*n* = 32, ∼24.1 years of age) and older adults (*n* = 120, ∼66.5 years of age) and found that aerobic fitness was associated with increased connectivity in the DMN, specifically between the PCC and medial prefrontal gyrus.^25^ Our results indicate that this association may also be applicable for persons with DS, with overall DMN connectivity being significantly associated with cardiorespiratory fitness even after adjustment for age. Given that there was also no association between MVPA and VO_2peak_ in this sample, this could indicate that fitness is more important than MVPA for DMN connectivity. The mechanism linking cardiorespiratory fitness and DMN connectivity is not fully understood but may be at least partially explained by increased cerebral blood flow,^43^ increased neurogenesis,^44^ or improved brain glucose metabolism^45^ associated with greater cardiorespiratory fitness but not MVPA. For example, cardiorespiratory fitness has been associated with efficient brain metabolism and cerebral oxygen utilization,^24^ which may impact brain aging. Evaluating the effect of both MVPA and cardiorespiratory fitness on cerebral blood flow using ASL would be important to confirm if this mechanism drives the outcomes seen in this analysis.

More work is needed to identify the effect of the duration of sustained fitness levels on these potential mechanisms identified and, ultimately, brain function. For example, in a sample of 120 older adults randomized to either aerobic training or a stretching control, Erickson et al. concluded that at 1 year, the aerobic training group increased hippocampal volume,^46^ which may impact DMN connectivity due to the role of the hippocampus in the DMN.^47^, ^48^ Similarly, in response to a 6‐month aerobic training program, 59 healthy older adults (age 60 to 79) saw increases in gray and white matter volume.^49^ In response to a single 30‐min bout of cycling, Weng et al. found an increase in synchrony in brain regions associated with learning and memory^50^; however, it is not clear how long lasting these acute increases are. So while acute exercise seems to provide immediate benefits to brain networks, sustained exercise over time may be necessary to drive changes in brain structure and function. In general, it is not known how long these physical activity habits or cardiorespiratory fitness need to be sustained to see benefits in the DMN in the general population or in populations with DS, which should be explored in the future.

The MPFC is involved with several cognitive processes, including executive functioning, working memory, and emotional regulation.^51^, ^52^ In adults without DS, studies have demonstrated weakened connectivity of the MPFC for those with mild cognitive impairment compared with non‐impaired controls.^53^, ^54^ Previous work also demonstrated that participants with high amyloid burden without any evident cognitive impairment reduced the connectivity of the MPFC than participants with low amyloid burden, suggesting that this reduced connectivity could indicate a preclinical stage of AD.^55^, ^56^ The present study found an association between cardiorespiratory fitness and FC of this brain region in persons with DS, which may implicate fitness as a potential protective factor against AD and cognitive decline for persons with DS. However, given the cross‐sectional nature and small sample size, it would be important to conduct larger and longitudinal studies investigating this association. Future work should investigate the association between MPFC connectivity and amyloid burden in persons with DS.

This study found no association between MVPA and DMN connectivity. This is in line with findings from Voss et al. (2016) in a sample of older adults (*n* = 189, average age 65 years), which concluded that FC of the DMN was positively associated with cardiorespiratory fitness but not habitual MVPA participation.^57^ These findings suggest that cardiorespiratory fitness may be more important for FC than MVPA engagement, given that there were associations between cardiorespiratory and FC. Studies have shown that determinants of cardiorespiratory fitness include many factors outside of physical activity participation, such as genetics.^58^ This could be why this study did not find an association between MVPA and cardiorespiratory fitness or MVPA and DMN connectivity in this sample.

Some research suggests that interventions increasing MVPA may benefit DMN connectivity. A randomized intervention in typical developed older adults (*n* = 65) compared the effect of a 12‐month aerobic training (walking) intervention versus a flexibility, toning, and balance intervention and observed that the aerobic training group increased the FC of several DMN nodes.^59^ However, it is unclear if this change in DMN connectivity was due to an increase in fitness or MVPA. Given that the increase was seen in the aerobic training arm, these findings suggest that specifically an increase in aerobic physical activity would be necessary to elicit changes in DMN connectivity. MVPA is defined as an activity reaching at least three metabolic equivalents (METs), which is the equivalent of walking at a moderate pace, and is unlikely to be achieved through flexibility or balance exercise. Currently, it is not known if increasing MVPA engagement could benefit DMN connectivity in persons with DS, which could be investigated in future interventions.

This study had several limitations, including the modest sample size and cross‐sectional analysis. Given that the MRI was an optional procedure for the parent study, a limited sample completed this assessment when compared to the size of the parent study (∼50%). Another limitation related to the imaging data is that a small proportion of the participants (9%, *n* = 3) did not have usable data physiological regressors, and their data were processed without this correction. The inclusion of these participants maximized the available sample size, and some work suggests that while subject‐level analysis benefits from such corrections, the group‐level impact of this correction is minor.^60^ Thus, with a small proportion of the sample not being corrected for this is unlikely to significantly bias the results. Still, future work would benefit from ensuring complete physiological recordings to enable uniform preprocessing across all participants. Other limitations include the sample was also primarily non‐Hispanic White, which limits the generalizability of the findings. Future work should ensure the inclusion of those of various races and ethnicities to understand the implications of these findings for those with various backgrounds. Additionally, this analysis did not account for other factors known to impact AD progression for adults with DS, including apolipoprotein E (APOE) genotyping. Since the parent study required participants to be sedentary, there was little variability in MVPA and VO_2Peak_ between participants; thus, future work should include participants across the spectrum of fitness and physical activity engagement to investigate these associations further. Notably, persons with DS report more sedentary behavior and less MVPA than populations without intellectual disability.^61^, ^62^ For the parent study, out of the 43 participants excluded from participating, 12 were excluded for reporting too much physical activity at baseline (∼27%). Still, this means there are a number of people with DS who are physically active and should be included in future work. It is not known whether the associations found in this paper would remain significant if a wider range of physical activity and fitness were included in the sample. Other exclusion criteria of the parent study, including those with poorer functional capacity (exclusion of those unable to walk 10 feet unassisted) and those unable to communicate through spoken language, could also limit the findings. These groups may be at higher risk for some of the decreases in connectivity and it would be important to include them in future work to understand a wider range of influence of fitness on connectivity. Finally, due to the exploratory nature of this analysis, being the first (to our knowledge) to evaluate the association between fitness and DMN FC, we did not adjust for multiple comparisons in our analyses. The priority of this analysis was to identify meaningful trends that could inform future studies for people with DS. Thus, there were no corrections for multiple comparisons, which could have increased the risk of Type I error.

There is some evidence that cardiorespiratory fitness may be associated with DMN connectivity in adults with DS. Future intervention trials should examine whether increasing cardiorespiratory fitness leads to changes in FC and, subsequently, cognition in adults with DS.

## CONFLICT OF INTEREST STATEMENT

J.G.C. receives funding from the NIH unrelated to this work. B.C.H. receives funding from the NIH unrelated to this work and receives funding for consulting for Special Olympics International. J.C.D., M.G.B. receive funding from the NIH related to this work. M.G.B. and J.R.S. receive funding from the NIH, both related and unrelated to this work. R.L. receives funding from the NIH unrelated to this work. L.M. receives funding from the NIH, both related and unrelated to this work. She has received honoria for a panel discussion for the National Center for Faculty Development and Diversity. J.E.D. receives funding from the NIH, both related and unrelated to this work. He receives funding for consulting from the University of Colorado and the University of Massachusetts, and funding from Health Partners (Minneapolis) for the Data Safety and Monitoring Board. L.T.P. receives funding from the NIH, both related and unrelated to this work, and research support to conduct clinical trials (paid to the institution) from ACI‐24. Author disclosures are available in Supporting Information.

## ETHICS APPROVAL

The parent trial was approved by the Institutional Review Board at the University of Kansas Medical Center. All participants provided written consent or legal guardian consent and written assent.

## Supporting information



Supporting Information

## Data Availability

Deidentified individual participant data (including data dictionaries) will be made available, as will study protocols, the statistical analysis plan, and the informed consent form, upon publication to researchers who provide a methodologically sound proposal for use in achieving the goals of the approved proposal. Proposals should be submitted to the corresponding author at jclina@kumc.edu.

## References

[1] Mai CT , Isenburg JL , Canfield MA , et al. National population‐based estimates for major birth defects, 2010‐2014. Birth Defects Res. 2019;111(18):1420‐1435. doi:10.1002/bdr2.1589 31580536 PMC7203968

[2] Englund A , Jonsson B , Zander CS , Gustafsson J , Annerén G . Changes in mortality and causes of death in the Swedish Down syndrome population. Am J Med Genet A. 2013;161(4):642‐649. 10.1002/ajmg.a.35706 23436430

[3] Antonarakis SE , Skotko BG , Rafii MS , et al. Down syndrome. Nat Rev Dis Primers. 2020;6(1):9. 32029743 10.1038/s41572-019-0143-7 PMC8428796

[4] De Graaf G , Buckley F , Skotko BG . Estimation of the number of people with Down syndrome in the United States. Genet Med. 2017;19(4):439‐447. 27608174 10.1038/gim.2016.127

[5] Zigman WB . Atypical aging in Down syndrome. Dev Disabil Res Rev. 2013;18(1):51‐67. 23949829 10.1002/ddrr.1128

[6] Hartley D , Blumenthal T , Carrillo M , et al. Down syndrome and Alzheimer's disease: common pathways, common goals. Alzheimers Dement. 2015;11(6):700‐709. 25510383 10.1016/j.jalz.2014.10.007 PMC4817997

[7] Fortea J , Vilaplana E , Carmona‐Iragui M , et al. Clinical and biomarker changes of Alzheimer's disease in adults with Down syndrome: a cross‐sectional study. Lancet. 2020;395(10242):1988‐1997. doi:10.1016/S0140-6736(20)30689-9 32593336 PMC7322523

[8] Wiseman FK , Al‐Janabi T , Hardy J , et al. A genetic cause of Alzheimer disease: mechanistic insights from Down syndrome. Nat Rev Neurosci. 2015;16(9):564. 26243569 10.1038/nrn3983 PMC4678594

[9] Lott IT . Antioxidants in Down syndrome. Biochim Biophys Acta, Mol Basis Dis. 2012;1822(5):657‐663. doi:10.1016/j.bbadis.2011.12.010 PMC3408054 22206998

[10] Mann DM . The pathological association between Down syndrome and Alzheimer disease. Mech Ageing Dev. 1988;43(2):99‐136. 2969441 10.1016/0047-6374(88)90041-3

[11] Rumble B , Retallack R , Hilbich C , et al. Amyloid A4 protein and its precursor in Down's syndrome and Alzheimer's disease. N Engl J Med. 1989;320(22):1446‐1452. doi:10.1056/nejm198906013202203 2566117

[12] Wisniewski KE , Wisniewski HM , Wen GY . Occurrence of neuropathological changes and dementia of Alzheimer's disease in Down's syndrome. Ann Neurol. 1985;17(3):278‐282. doi:10.1002/ana.410170310 3158266

[13] Iulita MF , Garzón Chavez D , Klitgaard Christensen M , et al. Association of Alzheimer disease with life expectancy in people with Down syndrome. JAMA Netw Open. 2022;5(5):e2212910‐e2212910. doi:10.1001/jamanetworkopen.2022.12910 35604690 PMC9127560

[14] Rosas HD , Lewis LR , Mercaldo ND , et al. Altered connectivity of the default mode network in cognitively stable adults with Down syndrome:“Accelerated aging” or a prelude to Alzheimer's disease? Alzheimer's Dement: Diagn Assess Dis Monit. 2021;13(1):e12105. 10.1002/dad2.12105 PMC8136300 34027014

[15] Weiler M , de Campos BM , Nogueira MH , Damasceno BP , Cendes F , Balthazar ML . Structural connectivity of the default mode network and cognition in Alzheimer׳ s disease. Psychiatry Res Neuroimaging. 2014;223(1):15‐22. 10.1016/j.pscychresns.2014.04.008 24816337

[16] Wu X , Li R , Fleisher AS , et al. Altered default mode network connectivity in alzheimer's disease—A resting functional MRI and bayesian network study. Hum Brain Mapp. 2011;32(11):1868‐1881. doi:10.1002/hbm.21153 21259382 PMC3208821

[17] Ereira S , Waters S , Razi A , Marshall CR . Early detection of dementia with default‐mode network effective connectivity. Nat Mental Health. 2024;2(7):787‐800. doi:10.1038/s44220-024-00259-5 PMC7618740 41716754

[18] DiProspero ND , Keator DB , Phelan M , et al. Selective impairment of long‐range default mode network functional connectivity as a biomarker for preclinical Alzheimer's disease in people with Down Syndrome. J Alzheimers Dis. 2022;85(1):153‐165. doi:10.3233/jad-210572 34776436 PMC9017677

[19] Law LL , Rol RN , Schultz SA , et al. Moderate intensity physical activity associates with CSF biomarkers in a cohort at risk for Alzheimer's disease. Alzheimer's Dement: Diagn Assess Dis Monit. 2018;10:188‐195. 10.1016/j.dadm.2018.01.001 PMC5842318 29527551

[20] Stephen R , Hongisto K , Solomon A , Lönnroos E . Physical activity and Alzheimer's disease: a systematic review. J Gerontol A. 2017;72(6):733‐739. doi:10.1093/gerona/glw251 28049634

[21] Hayes SM , Hayes JP , Cadden M , Verfaellie M . A review of cardiorespiratory fitness‐related neuroplasticity in the aging brain. Front Aging Neurosci. 2013;5:31. 23874299 10.3389/fnagi.2013.00031 PMC3709413

[22] Rovio S , Kåreholt I , Helkala E‐L , et al. Leisure‐time physical activity at midlife and the risk of dementia and Alzheimer's disease. Lancet Neurol. 2005;4(11):705‐711. doi:10.1016/S1474-4422(05)70198-8 16239176

[23] Boraxbekk C‐J , Salami A , Wåhlin A , Nyberg L . Physical activity over a decade modifies age‐related decline in perfusion, gray matter volume, and functional connectivity of the posterior default‐mode network—A multimodal approach. Neuroimage. 2016;131:133‐141. doi:10.1016/j.neuroimage.2015.12.010 26702778

[24] Olivo G , Nilsson J , Garzón B , et al. Higher VO2max is associated with thicker cortex and lower grey matter blood flow in older adults. Sci Rep. 2021;11(1):16724. 34408221 10.1038/s41598-021-96138-5 PMC8373929

[25] Voss MW , Erickson KI , Prakash RS , et al. Functional connectivity: a source of variance in the association between cardiorespiratory fitness and cognition?. Neuropsychologia. 2010;48(5):1394‐1406. doi:10.1016/j.neuropsychologia.2010.01.005 20079755 PMC3708614

[26] Ptomey LT , Szabo‐Reed AN , Martin LE , et al. The promotion of physical activity for the prevention of Alzheimer's disease in adults with Down Syndrome: rationale and design for a 12‐month randomized trial. Contemp Clin Trials Commun. 2020;19:100607. 32642594 10.1016/j.conctc.2020.100607 PMC7334572

[27] Deb S , Hare M , Prior L , Bhaumik S . Dementia screening questionnaire for individuals with intellectual disabilities. Br J Psychiatry: J Mental Sci. 2007;190:440‐444. doi:10.1192/bjp.bp.106.024984 17470960

[28] Choi L , Liu Z , Matthews CE , Buchowski MS . Validation of accelerometer wear and nonwear time classification algorithm. Med Sci Sports Exerc. 2011;43(2):357. 20581716 10.1249/MSS.0b013e3181ed61a3 PMC3184184

[29] Masse LC , Fuemmeler BF , Anderson CB , et al. Accelerometer data reduction: a comparison of four reduction algorithms on select outcome variables. Med Sci Sports Exercise. 2005;37(11):S544‐S554. 10.1249/01.mss.0000185674.09066.8a 16294117

[30] Troiano RP , Berrigan D , Dodd KW , Masse LC , Tilert T , McDowell M . Physical activity in the United States measured by accelerometer. Med Sci Sports Exerc. 2008;40(1):181‐188. doi:10.1249/mss.0b013e31815a51b3 18091006

[31] Matthews CE , Chen KY , Freedson PS , et al. Amount of time spent in sedentary behaviors in the United States, 2003‐2004. Am J Epidemiol. 2008;167(7):875‐881. 18303006 10.1093/aje/kwm390 PMC3527832

[32] Fernhall B , McCubbin JA , Pitetti KH , et al. Prediction of maximal heart rate in individuals with mental retardation. Med Sci Sports Exerc. 2001;33(10):1655‐1660. 11581548 10.1097/00005768-200110000-00007

[33] Kim SG , Uĝurbil K . Comparison of blood oxygenattion and cerebral blood flow effect in fMRI: estimation of relative oxygen consumption change. Magn Reson Med. 1997;38(1):59‐65. 9211380 10.1002/mrm.1910380110

[34] Tetzlaff JE , Huppenbauer CB , Tanzer L , Alexander TD , Jones KJ . Motoneuron injury and repair: new perspectives on gonadal steroids as neurotherapeutics. J Mol Neurosci. 2006;28:53‐64. 16632875 10.1385/jmn:28:1:53

[35] Moeller S , Yacoub E , Olman CA , et al. Multiband multislice GE‐EPI at 7 tesla, with 16‐fold acceleration using partial parallel imaging with application to high spatial and temporal whole‐brain fMRI. Magn Reson Med. 2010;63(5):1144‐1153. doi:10.1002/mrm.22361 20432285 PMC2906244

[36] Feinberg DA , Moeller S , Smith SM , et al. Multiplexed echo planar imaging for sub‐second whole brain FMRI and fast diffusion imaging. PLoS One. 2010;5(12):e15710. doi:10.1371/journal.pone.0015710 21187930 PMC3004955

[37] Xu J , Moeller S , Auerbach EJ , et al. Evaluation of slice accelerations using multiband echo planar imaging at 3 T. Neuroimage. 2013;83:991‐1001. doi:10.1016/j.neuroimage.2013.07.055 23899722 PMC3815955

[38] Cox RW . AFNI: software for analysis and visualization of functional magnetic resonance neuroimages. Comput Biomed Res. 1996;29(3):162‐173. 8812068 10.1006/cbmr.1996.0014

[39] Lepping RJ , Yeh H‐W , McPherson BC , et al. Quality control in resting‐state fMRI: the benefits of visual inspection. Front Neurosci. 2023;17:1076824. 37214404 10.3389/fnins.2023.1076824 PMC10192849

[40] Whitfield‐Gabrieli S , Nieto‐Castanon A . Conn: a functional connectivity toolbox for correlated and anticorrelated brain networks. Brain connectivity. 2012;2(3):125‐141. 22642651 10.1089/brain.2012.0073

[41] R Core Team . R: A Language and Environment for Statistical Computing. R Foundation for Statistical Computing; 2022.

[42] Kronman CA , Kern KL , Nauer RK , Dunne MF , Storer TW , Schon K . Cardiorespiratory fitness predicts effective connectivity between the hippocampus and default mode network nodes in young adults. Hippocampus. 2020;30(5):526‐541. 31647603 10.1002/hipo.23169 PMC7442492

[43] Brown AD , McMorris CA , Longman RS , et al. Effects of cardiorespiratory fitness and cerebral blood flow on cognitive outcomes in older women. Neurobiol Aging. 2010;31(12):2047‐2057. 19111937 10.1016/j.neurobiolaging.2008.11.002

[44] Erickson KI , Gildengers AG , Butters MA . Physical activity and brain plasticity in late adulthood. Dialogues Clin Neurosci. 2013;15(1):99‐108. doi:10.31887/DCNS.2013.15.1/kerickson 23576893 PMC3622473

[45] Gaitán JM , Boots EA , Dougherty RJ , et al. Brain glucose metabolism, cognition, and cardiorespiratory fitness following exercise training in adults at risk for Alzheimer's Disease. Brain Plasticity. 2019;5:83‐95. doi:10.3233/BPL-190093 31970062 PMC6971821

[46] Erickson KI , Voss MW , Prakash RS , et al. Exercise training increases size of hippocampus and improves memory. Proc Natl Acad Sci. 2011;108(7):3017‐3022. 21282661 10.1073/pnas.1015950108 PMC3041121

[47] Aberizk K , Sefik E , Addington J , et al. Hippocampal connectivity with the default mode network is linked to hippocampal volume in the clinical high risk for psychosis syndrome and healthy individuals. Clin Psychol Sci. 2023;11(5):801‐818. doi:10.1177/21677026221138819 37981950 PMC10656030

[48] Bassett DS , Sporns O . Network neuroscience. Nat Neurosci. 2017;20(3):353‐364. 28230844 10.1038/nn.4502 PMC5485642

[49] Colcombe SJ , Erickson KI , Scalf PE , et al. Aerobic exercise training increases brain volume in aging humans. J Gerontol A Biol Sci Med Sci. 2006;61(11):1166‐1170. doi:10.1093/gerona/61.11.1166 17167157

[50] Weng TB , Pierce GL , Darling WG , Falk D , Magnotta VA , Voss MW . The acute effects of aerobic exercise on the functional connectivity of human brain networks. Brain Plasticity. 2016;2(2):171‐190. 10.3233/BPL-160039 PMC5928541 29765855

[51] Jobson DD , Hase Y , Clarkson AN , Kalaria RN . The role of the medial prefrontal cortex in cognition, ageing and dementia. Brain Commun. 2021;3(3):fcab125. doi:10.1093/braincomms/fcab125 34222873 PMC8249104

[52] Xu P , Chen A , Li Y , Xing X , Lu H . Medial prefrontal cortex in neurological diseases. Physiol Genomics. 2019;51(9):432‐442. doi:10.1152/physiolgenomics.00006.2019 31373533 PMC6766703

[53] Yue C , Wu D , Bai F , et al. State‐based functional connectivity changes associate with cognitive decline in amnestic mild cognitive impairment subjects. Behav Brain Res. 2015;288:94‐102. doi:10.1016/j.bbr.2015.04.013 25907751

[54] Cai S , Chong T , Peng Y , et al. Altered functional brain networks in amnestic mild cognitive impairment: a resting‐state fMRI study. Brain Imaging Behav. 2017;11(3):619‐631. doi:10.1007/s11682-016-9539-0 26972578

[55] Sheline YI , Raichle ME , Snyder AZ , et al. Amyloid plaques disrupt resting state default mode network connectivity in cognitively normal elderly. Biol Psychiatry. 2010;67(6):584‐587. doi:10.1016/j.biopsych.2009.08.024 19833321 PMC2829379

[56] Hedden T , Van Dijk KRA , Becker JA , et al. Disruption of functional connectivity in clinically normal older adults harboring amyloid burden. J Neurosci. 2009;29(40):12686. doi:10.1523/JNEUROSCI.3189-09.2009 19812343 PMC2808119

[57] Voss MW , Weng TB , Burzynska AZ , et al. Fitness, but not physical activity, is related to functional integrity of brain networks associated with aging. Neuroimage. 2016;131:113‐125. doi:10.1016/j.neuroimage.2015.10.044 26493108 PMC4837101

[58] Bouchard C , Sarzynski MA , Rice TK , et al. Genomic predictors of the maximal O2 uptake response to standardized exercise training programs. J Appl Physiol. 2011;110(5):1160‐1170. 21183627 10.1152/japplphysiol.00973.2010 PMC3098655

[59] Voss MW , Prakash RS , Erickson KI , et al. Plasticity of brain networks in a randomized intervention trial of exercise training in older adults. Front Aging Neurosci. 2010;2:32. doi:10.3389/fnagi.2010.00032 20890449 PMC2947936

[60] Starck T , Remes J , Nikkinen J , Tervonen O , Kiviniemi V . Correction of low‐frequency physiological noise from the resting state BOLD fMRI—Effect on ICA default mode analysis at 1.5T. J Neurosci Methods. 2010;186(2):179‐185. doi:10.1016/j.jneumeth.2009.11.015 19941896

[61] Siaplaouras J , Niessner C , Helm PC , et al. Physical activity among children with congenital heart defects in Germany: a nationwide survey. Front Pediatr. 2020;8:170. 32426306 10.3389/fped.2020.00170 PMC7203217

[62] Dua JS , Cooper AR , Fox KR , Stuart AG . Physical activity levels in adults with congenital heart disease. Eur J Cardiovasc Prev Rehabil. 2007;14(2):287‐293. 17446809 10.1097/HJR.0b013e32808621b9

